# Amyotrophic lateral sclerosis and osteoporosis: a two-sample Mendelian randomization study

**DOI:** 10.3389/fnagi.2023.1305040

**Published:** 2023-12-14

**Authors:** Junhong Li, Cong Ma, Hui Huang, Hui Liao

**Affiliations:** Department of Orthopedics, Tongji Hospital, Tongji Medical College, Huazhong University of Science and Technology, Wuhan, China

**Keywords:** amyotrophic lateral sclerosis, osteoporosis, Mendelian randomization, causality, bone mineral density

## Abstract

**Background:**

A few observational studies revealed that amyotrophic lateral sclerosis (ALS) was tightly connected with osteoporosis. However, the results of previous studies were inconsistent, and the causal effect of ALS on osteoporosis has not been investigated. To do so, the two-sample Mendelian randomization (MR) method was employed to estimate the causality.

**Methods:**

The instrumental variables (IVs) for ALS were selected from one GWAS summary dataset (27,205 ALS cases and 110,881 controls), and bone mineral density (BMD) in the femoral neck (FN), lumbar spine (LS), and forearm, extracted from another large-scale GWAS summary database (53,236 cases), were used as phenotypes for osteoporosis. Random-effects inverse variance weighted (IVW), MR Egger, weighted median, simple mode, and weighted mode were conducted to evaluate the causality. Sensitivity analyses were further performed to explore heterogeneity and pleiotropy.

**Results:**

A total of 10 qualified SNPs were finally selected as proxies for ALS. The results of random effects from IVW revealed that ALS has no causal effect on FN-BMD (beta: −0.038, 95% CI: −0.090 to 0.015, SE: 0.027, *p* = 0.158), LS-BMD (beta: −0.015, 95% CI: −0.076 to 0.046, SE: 0.031, *p* = 0.629), and forearm BMD (beta: 0.044, 95% CI: −0.063 to 0.152, SE: 0.055, *p* = 0.418). These results were confirmed using the MR-Egger, weighted median, simple model, and weighted model. No heterogeneity or pleiotropy was detected (*p* > 0.05 for all).

**Conclusion:**

Contrary to previous observational studies, our study figured out that no causal effect existed between ALS and osteoporosis. The disparity in results is probably attributed to secondary effects such as physical inactivity and muscle atrophy caused by ALS.

## 1 Introduction

Both amyotrophic lateral sclerosis (ALS) and osteoporosis are age-related diseases that can severely exacerbate the debilitation of the musculoskeletal system (Pandya and Patani, 2020; Ma et al., 2023). ALS is a rare, fatal, and incurable disorder characterized by motor neuron dysfunction leading to progressive skeletal muscle weakness and behavioral deficits, with respiratory failure often the ultimate cause of death (Feldman et al., 2022). Osteoporosis is a generalized systemic skeletal disorder associated with decreased bone mass and disruption of bone architecture, resulting in a subsequent increase in susceptibility to fractures (including hip, spine, and pelvic fractures) (Genant et al., 1999). Numerous clinical studies have observed that the skeletal health of ALS patients significantly deteriorates due to a lack of muscle contraction and physical activity, manifested as an increased risk of bone loss and osteoporosis (Brown and Al-Chalabi, 2017; Caplliure-Llopis et al., 2020). According to statistics, the annual incidence of osteoporotic fractures caused by osteoporosis exceeds 8.9 million cases worldwide, most of which usually require long-term care, and the mortality rate of patients with a disease duration of more than 1 year is increased by 25%, thereby causing unoptimistic treatment costs and mortality (Ferrari et al., 2016; Ensrud and Crandall, 2017; Johnston and Dagar, 2020). Nonetheless, the problem of comorbid osteoporosis in ALS patients is always underestimated, as ALS patients often have limited mobility and bone loss tends to occur insidiously (usually asymptomatic until the first osteoporotic fracture), which poses a challenge for clinical management and intervention (Johnston and Dagar, 2020; Aibar-Almazán et al., 2022). Non-invasive mechanical ventilation (NIMV) contributes to the prolonged survival of ALS patients, yet growing clinical cases have reported that ALS patients treated with NIMV who are comorbid with severe osteoporosis are more susceptible to respiratory failure due to a low-energy traumatic event that induces osteoporotic vertebral fracture (Hardiman et al., 2017; Portaro et al., 2018). Evaluating the correlation between ALS and osteoporosis, as well as timely identifying and treating ALS patients with the potential risk of osteoporosis and fracture, are bound to be of great clinical importance.

Previous studies have suggested that there is an interacting pathophysiological basis and a close clinical correlation between ALS and osteoporosis (Medinas et al., 2017; Caplliure-Llopis et al., 2020; Ma et al., 2023). Pathophysiological studies have revealed that bone is a key provider of muscle trophic factors (e.g., BMP, VEGF, and IGF-1), whereas muscle is a source of osteoblastic stem cells and certain anabolic stimuli for bone remodeling (Cho et al., 2010; Koh et al., 2012; Zhou et al., 2015). Muscle strength continuously regulates the structure and function of the skeleton, so when ALS causes muscle dysfunction, the skeleton is compromised accordingly; likewise, compromised bone balance can, in turn, contribute to muscle degeneration, which further accelerates ALS progression (Zhou et al., 2015). Clinical studies have found that ALS patients are at increased risk of developing osteoporosis and related fractures because of abnormal energy metabolism, malnutrition, decreased limb flexibility, increased joint stiffness, and frequent falls (Fernando et al., 2019; Morini et al., 2023). According to multiple observational studies, there is a strong statistical correlation between ALS and osteoporosis, whereas it is difficult to state the exact nature of the relationship since these studies are observational (Joyce et al., 2012; Kelly et al., 2020; Sooragonda et al., 2021). Notably Caplliure-Llopis et al. (2020) indicated that deterioration in bone health was not associated with ALS subtype or clinical status but could be related to the levels of metabolic parameters like thyroid-stimulating hormone and vitamin D. Thus, the aforementioned conflicting evidence may weaken the potential causal relationship between these two diseases. It should be recognized that in epidemiological studies (especially observational studies), studies were commonly limited to small sample sizes (due to the rarity of ALS), and the presence of bias introduced by confounders largely interfered with the causal-effect inference of exposure and outcome, thereby rendering the results unreliable (Fewell et al., 2007). Further research focused on the causal relationship between ALS and osteoporosis would be valuable for the prevention and treatment of combined osteoporosis in ALS patients.

Mendelian randomization (MR) is an emerging method in clinical epidemiology that utilizes genetic variants as instrumental variables (IVs) and thus cannot be affected by confounding factors and reverse causation, which exist in cross-sectional studies (Davey Smith and Hemani, 2014). Based on this, the MR method has the ability to identify causal relationships between exposure and outcome and is also regarded as an alternative method for randomized controlled trials, which have been regarded as the gold standard for verifying causality (Davey Smith and Hemani, 2014; Davies et al., 2018). Owing to its strength of causal-effect inference, the two-sample MR method was widely used to find risk factors for a variety of diseases, such as osteoporosis, Parkinson’s disease, and prostate cancer (Mitchell et al., 2021; Bottigliengo et al., 2022; Deng and Wong, 2023; Fang et al., 2023). At the same time, a number of close relationships identified by previous cross-sectional studies were further validated and disentangled by recent MR studies (Chen et al., 2022; Zhang et al., 2022). For instance, previous observational studies have found that rheumatoid arthritis is closely related to osteoporosis, while through a two-sample MR analysis, Liu et al. (2021) demonstrated no causal association between rheumatoid arthritis and osteoporosis. Similarly, based on the results of MR analysis, He et al. (2021) did not find a causal relationship between depression and osteoporosis, which is contrary to the results of previous observational research. Unfortunately, to the best of our knowledge, there is still a lack of MR studies exploring the causal relationship between ALS and osteoporosis to validate the results of previously controversial observational studies and to provide guidance for future clinical interventions in ALS patients with comorbid osteoporosis.

The current study, as the first two-sample MR study to explore the causal effect of ALS on osteoporosis, ultimately aims to elucidate the causal relationship between ALS and osteoporosis and to corroborate the findings identified by previous cross-sectional studies.

## 2 Materials and methods

### 2.1 Data on ALS and BMD

The largest-scale ALS GWAS summary statistics up to now were extracted from a recent meta-analysis performed by van Rheenen et al. (2021), which involved 27,205 ALS cases and 110,881 controls. All participants were of European ancestry. For the ALS and BMD data sets used in this study (Zheng et al., 2015; van Rheenen et al., 2021), we refer the reader to the primary GWAS manuscripts and their Supplementary material for details on information of cohorts. Cases were diagnosed in accordance with the revised El-Escorial criteria (Brooks et al., 2000), and control subjects were population-based controls matched for sex and age. The summary-level data on ALS among European ancestry is publicly available through the Project MinE website.^1^

In the present study, we aimed to explore the causal effect of ALS on osteoporosis. Osteoporosis was diagnosed clinically by measurement of BMD to a large extent (LeBoff et al., 2022). BMD measured at three common bone areas, including femoral neck BMD (FN-BMD), lumbar spine (L1-4) BMD (LS-BMD), and the forearm (distal 1/3 of radius) BMD, were treated as outcome variables in the MR analysis. GWAS summary data for BMD were retrieved from a large meta-analysis involving a total of 53,236 cases of European ancestry, which were selected from the general population (Zheng et al., 2015). Sex, age, age-squared, and weight were covariates in the meta-analysis and adjusted before testing SNPs. BMD was measured by DXA and standardized within each cohort to control systematic differences in BMD measurements. The summary-level data of FN-BMD, LS-BMD, and forearm BMD used in this study were extracted from the GEnetic Factors for OSteoporosis Consortium (GEFOS, http://www.gefos.org/).

Population stratification was regarded as a factor that could contribute to bias in MR analysis caused by different ancestries between exposure and outcome summary statistics. In our study, all the participants were of European ancestry. In addition, the degree of sample overlap was an important factor to consider and could introduce bias or a type 1 error rate if it was substantial in a two-sample MR study (Burgess et al., 2016). We tested the degree through the online tool^2^ (Burgess et al., 2016), and no significant sample overlap (<6%) between exposure (ALS) participants and outcome (FN-BMD, LS-BMD, and forearm BMD) participants was found. A large study population, relatively strong IVs, and low sample overlap in this study could minimize the extent of bias caused by overlapped populations to some extent (Burgess et al., 2016). On account of all the GWAS summarized statistics of ALS and BMD used in the present study being publicly available, no ethical consent was needed.

### 2.2 IVs selection process

To obtain robust results in the MR analysis, MR was required to satisfy the three assumptions as follows: first, the relevance assumption: IVs were strongly associated with ALS in this study; second, the exclusion restriction assumption: IVs could only exert influence on BMD through ALS rather than other pleiotropic pathways; and third, the independence assumption: IVs were independent of confounders (Skrivankova et al., 2021). Accordingly, the SNPs of exposure data that satisfied the strict criteria as follows were employed as IVs. First, SNPs with *p*-value lower than 5 × 10^−8^ and *F*-statistics greater than 10 were regarded as significantly associated with the exposure factor and included in the study. The formula *F* = *R*^2^ (*N* − 2)/(1 − *R*^2^) and *R*^2^ = 2 × (1 − MAF) × MAF × *β*^2^ were used to calculate the strength of every single SNP. N represented the sample size of the ALS GWAS database, and MAF represented minor allele frequency. Second, the clumping process (*r*^2^ < 0.001, kb = 10,000) among all the above-included SNPs was carried out to exclude SNPs that were in linkage disequilibrium (LD) with other SNPs. *r*^2^ was the LD correlation coefficient. Third, if a large portion of SNPs were not found in the GWAS datasets of BMD, variant proxies (*r*^2^ ≥ 0.8) were selected by visiting the online website^3^ (Li et al., 2022; Seyedsalehi et al., 2023). Fourth, PhenoScanner V2 (available at http://www.phenoscanner.medschl.cam.ac.uk/) is a simple and widely used tool to specify whether a particular SNP is associated with other confounders (Kamat et al., 2019). SNPs related to confounders were excluded by visiting this online website. Previous studies have demonstrated that age at menarche has a causal effect on osteoporosis (Zhang et al., 2018). The SNP rs2077492 was excluded due to its strong association with age at menarche. The SNP rs9275477 was also excluded due to its association with treatment with prednisolone. Finally, SNPs that were significantly (*p*-value lower than 5 × 10^−8^) associated with outcome data were excluded. Incompatible SNPs were also excluded from the harmonization process. Finally, 10 SNPs of ALS were used as the IVs for further MR analysis. The flowchart of this study design was shown in Supplementary Figure S1.

### 2.3 Mendelian randomization analyses and sensitivity analyses

The causal effects of ALS on osteoporosis risk, including FN-BMD, LS-BMD, and forearm BMD, were estimated using the two-sample MR method. Random effects inverse variance weighted (IVW) was the main analytical method. Other complementary methods, including MR-Egger regression, weighted median, simple model, and weighted model, were also used.

The following sensitivity analyses were applied to explore heterogeneity and pleiotropy. Cochran’s *Q* tests using IVW and MR-Egger were calculated to estimate the heterogeneity. MR-Egger intercept analysis was used to detect the horizontal pleiotropic effects. *p*-values less than 0.05 indicated pleiotropy. A leave-one-out cross-validation test was applied to detect the effect of potentially influential SNPs and the robustness of the estimates. The MR pleiotropy residual sum and outlier test (MR-PRESSO) was conducted to test pleiotropy and detect and correct the outliers. If there were any outlier SNPs, the MR analysis was performed again after the outlier was corrected. The symmetry of the funnel plot could intuitively reflect potential horizontal pleiotropy, and any asymmetry was a sign of directional pleiotropy (Burgess et al., 2017). Statistical power was tested according to the method proposed by Brion et al. (2013). A *p*-value less than 0.05 indicated statistical significance in all the sensitivity analysis methods.

### 2.4 Statistical analysis

Package “TwoSampleMR” (Hemani et al., 2018) in the R software (version 4.1.2) was employed for two-sample MR analysis. Tests were two-tailed. When the number of tests is greater than one, Bonferroni correction should be performed to avoid false-positive estimates (Peng et al., 2022). Bonferroni-corrected statistical significance was 0.05/(1 ALS × 3 BMD) = 0.017 in this study. *p*-value less than 0.017 was considered significant in the MR analysis. *p*-value less than 0.05 but more than 0.017 was considered a potential causal relationship.

## 3 Results

After strict adherence to the criteria of IVs selection, a total of 10 qualified SNPs were finally selected as proxies for ALS in the present study (Supplementary Table S1). No proxy SNPs were employed because all the SNPs identified in the exposure data were found in the outcome data.

### 3.1 Causal effect of ALS on FN-BMD

**Figure 1:**
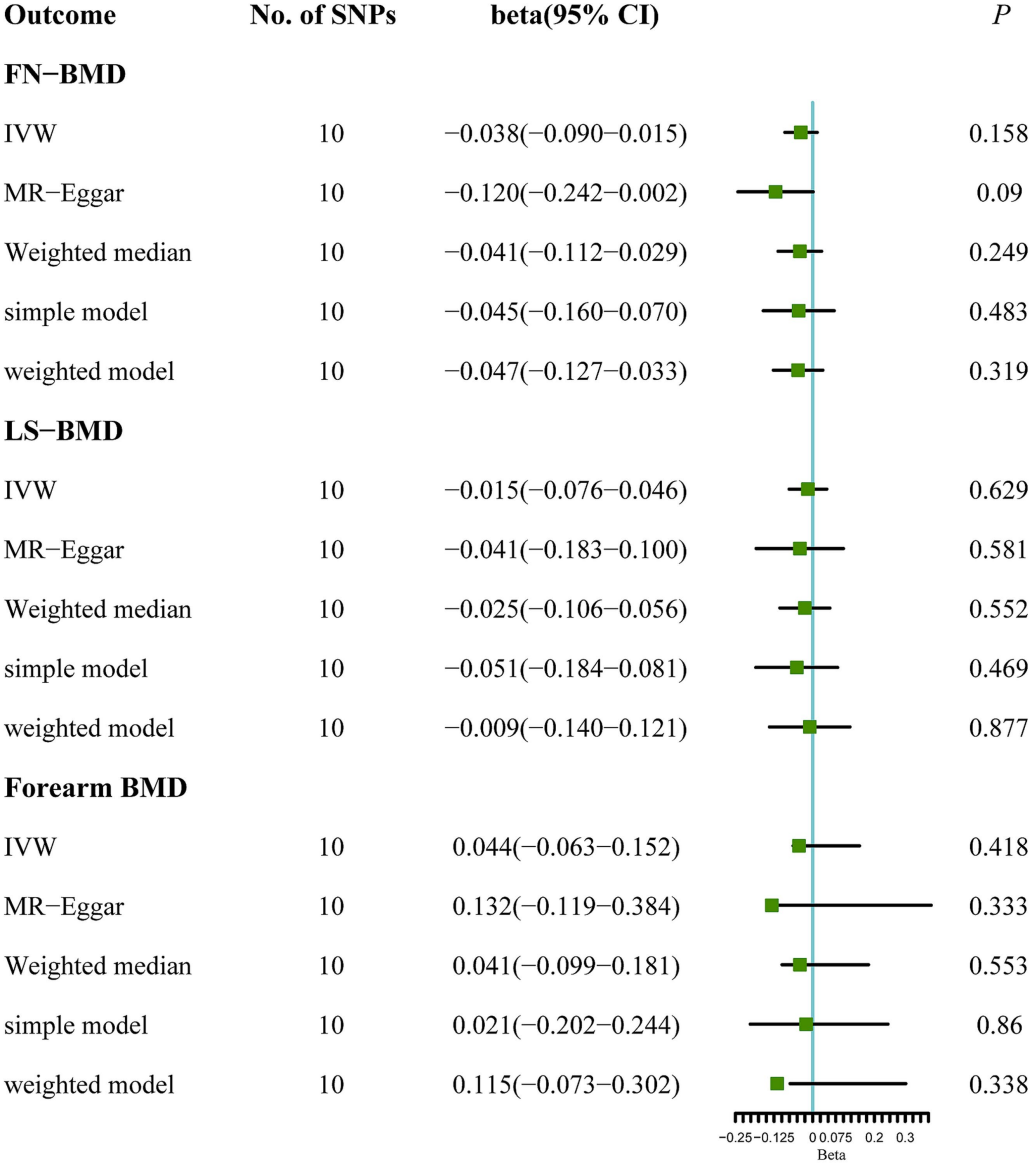
MR analysis between ALS and osteoporosis (FN-BMD, LS-BMD, and forearm BMD). Five methods: random-effects IVW, MR Egger, weighted median, simple mode, and weighted mode.

**Figure 2:**
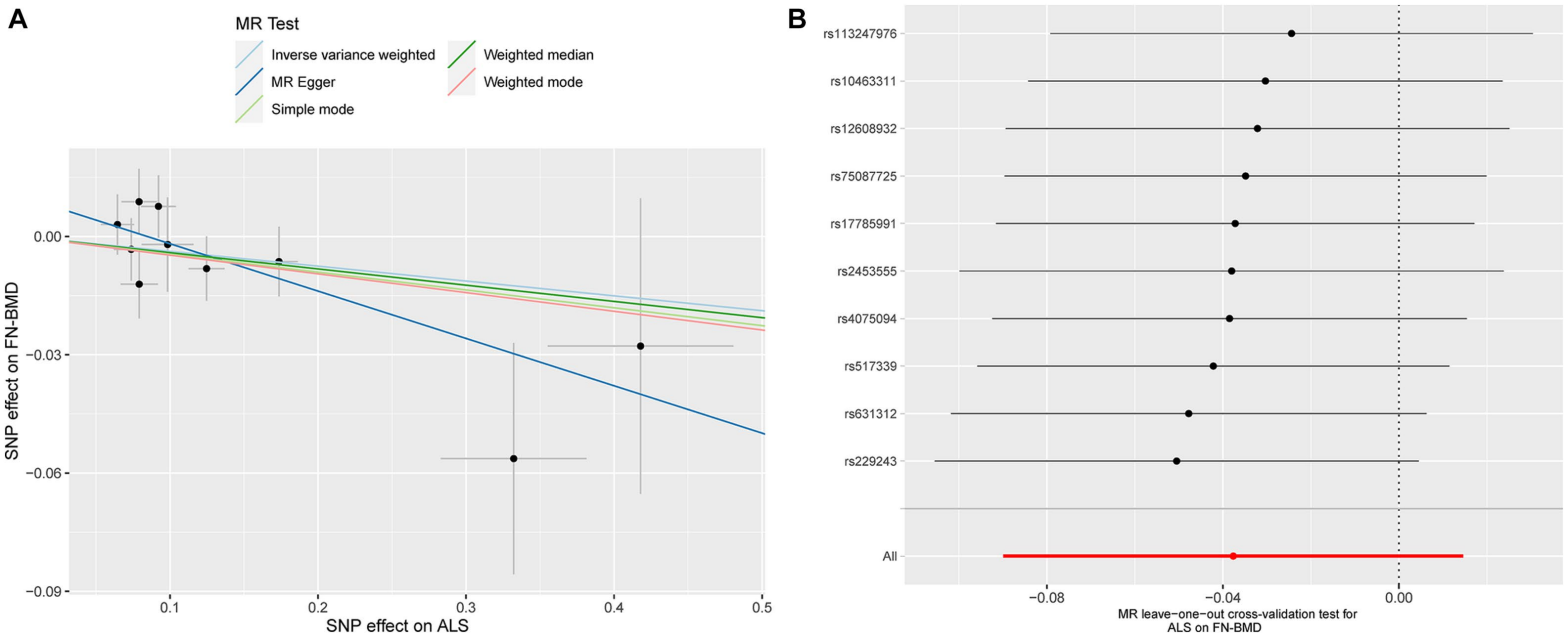
Scatterplot of the causal relationships between ALS and FN-BMD **(A)**. MR Leave-one-out cross-validation test for ALS on FN-BMD **(B)**.

Based on the results of the main MR method, ALS had no etiological effect on FN-BMD in IVW [beta: −0.038, 95% confidence interval (CI): −0.090 to 0.015, standard error (SE): 0.027, *p* = 0.158, Figure 1]. The results were validated in other complementary methods, MR-Egger (beta: −0.120, 95% CI: −0.242 to 0.002, SE: 0.062, *p* = 0.090), weighted median (beta: −0.041, 95% CI: −0.112 to 0.029, SE: 0.036, *p* = 0.249), simple model (beta: −0.045, 95% CI: −0.160 to 0.070, SE: 0.059, *p* = 0.483), and weighted model (beta: −0.047, 95% CI: −0.127 to 0.033, SE: 0.041, *p* = 0.319, Figure 1). A scatterplot of the causal associations between ALS and FN-BMD is shown in Figure 2A.

### 3.2 Causal effect of ALS on LS-BMD

**Figure 3:**
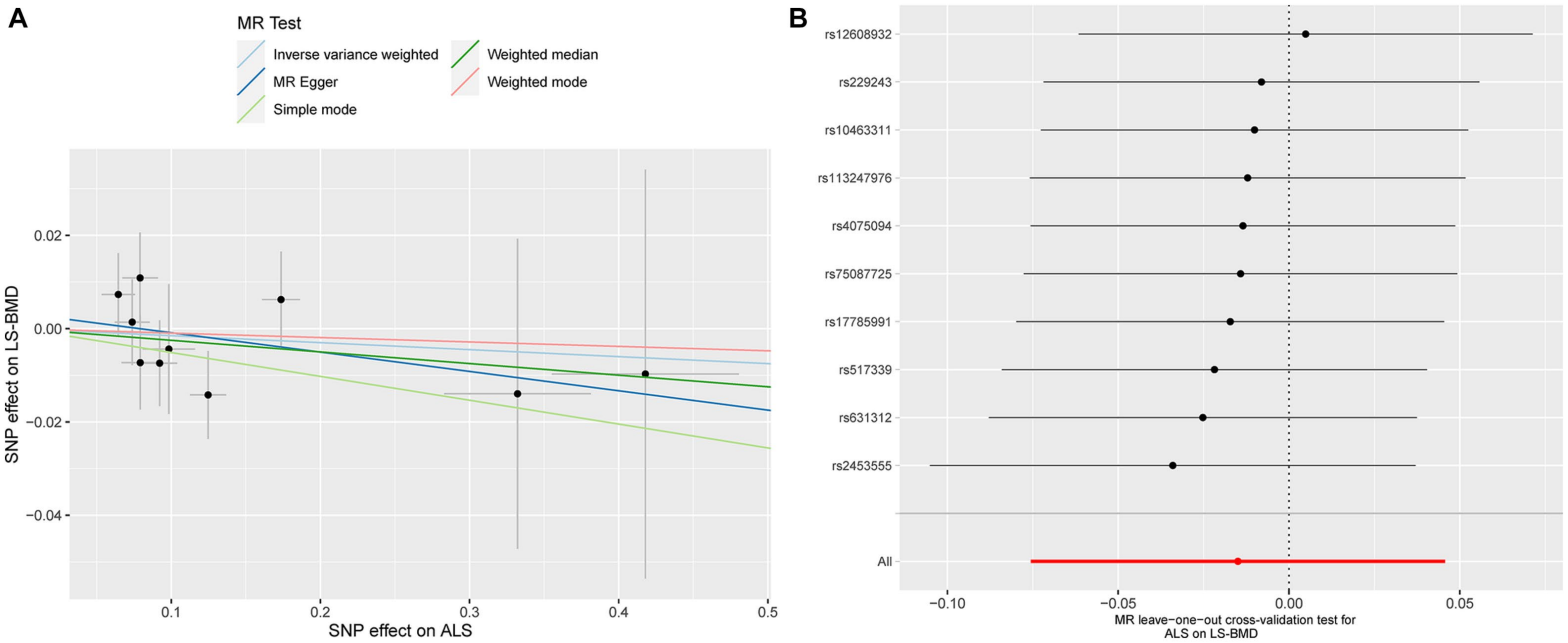
Scatterplot of the causal relationships between ALS and LS-BMD **(A)**. MR Leave-one-out cross-validation test for ALS on LS-BMD **(B)**.

Similarly, no significant causal effect was identified between ALS and LS-BMD, estimated by the following five two-sample MR analysis methods (IVW beta: −0.015, 95% CI: −0.076 to 0.046, SE: 0.031, *p* = 0.629; MR-Egger beta: −0.041, 95% CI: −0.183 to 0.100, SE: 0.072, *p* = 0.581; weighted median beta: −0.025, 95% CI: −0.106 to 0.056, SE: 0.041, *p* = 0.552; simple model beta: −0.051, 95% CI: −0.184 to 0.081, SE: 0.068, *p* = 0.469; weighted model beta: −0.009, 95% CI: −0.140 to 0.121, SE: 0.067, *p* = 0.877, Figure 1). The forest map of the above five MR methods is shown in Figure 1. A scatterplot of the causal relationships between ALS and LS-BMD is shown in Figure 3A.

### 3.3 Causal effect of ALS on forearm BMD

**Figure 4:**
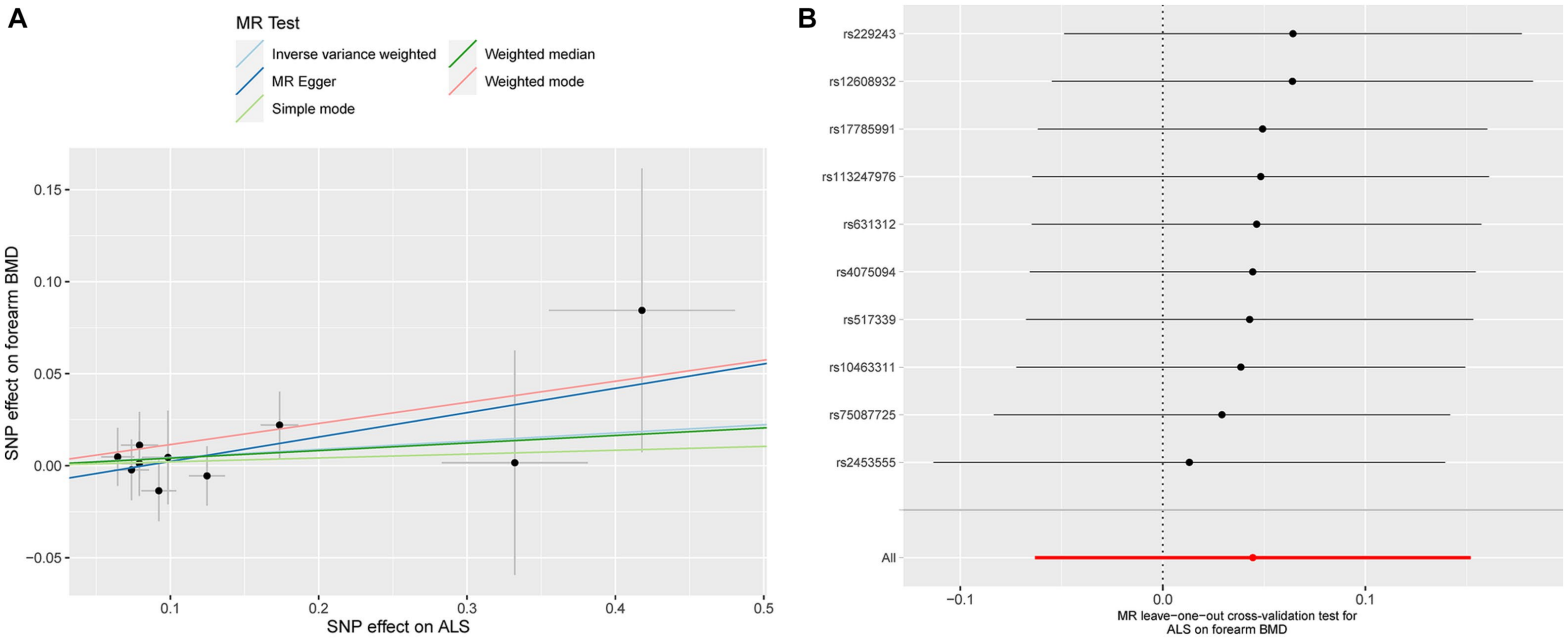
Scatterplot of the causal relationships between ALS and forearm BMD **(A)**. MR Leave-one-out cross-validation test for ALS on forearm BMD **(B)**.

ALS also showed a null causal relationship with forearm BMD in IVW (beta: 0.044, 95% CI: −0.063 to 0.152, SE: 0.055, *p* = 0.418), MR-Egger (beta: 0.132, 95% CI: −0.119 to 0.384, SE: 0.128, *p* = 0.333), weighted-median (beta: 0.041, 95% CI: −0.099 to 0.181, SE: 0.072, *p* = 0.553), simple model (beta: 0.021, 95% CI: −0.202 to 0.244, SE: 0.114, *p* = 0.860), and weighted model (beta: 0.115, 95% CI: −0.073 to 0.302, SE: 0.096, *p* = 0.338, Figure 1). The scatterplot of the causal associations between ALS and forearm BMD is shown in Figure 4A.

### 3.4 Sensitivity analyses

Detailed results of MR-PRESSO, MR-Egger intercept analysis, heterogeneity tests by IVW, and heterogeneity tests by MR-Egger are shown in Table 1. All the *p*-values were greater than 0.05 in MR-Egger intercept analysis, heterogeneity tests by IVW, and heterogeneity tests by MR-Egger, indicating that no heterogeneity or pleiotropy existed in our study. In addition, no outliers were identified by MR-PRESSO analysis. According to the results of the leave-one-out cross-validation test, we did not detect any potentially influential or problematic SNPs (Figures 2B–4B). As shown in the funnel plots (Supplementary Figure S2), no potential horizontal pleiotropy was discovered. Based on the consistency of the results estimated by five different MR analytical approaches and the fact that no positive findings existed in the heterogeneity and pleiotropy tests, the results were considered robust.

**Table 1:** The results of sensitivity analyses.

Exposure	Outcome	MR-PRESSO		MR-Egger intercept analysis		Heterogeneity tests by IVW		Heterogeneity tests by MR-Egger	
		Global test *p*-value	Main MR results *p*-value	Intercept	Intercept *p*-value	Cochran’s *Q*	*p*-value	Cochran’s *Q*	*p*-value
ALS	FN-BMD	0.570	0.171	0.010	0.180	8.120	0.522	5.967	0.651
ALS	LS-BMD	0.725	0.563	0.003	0.694	5.842	0.756	5.676	0.683
ALS	Forearm BMD	0.930	0.217	−0.011	0.470	3.342	0.949	2.768	0.948

*p* < 0.05 was considered significant in sensitivity analyses.

## 4 Discussion

As far as we know, this is the first study to estimate the genetic causal effect of ALS on osteoporosis by utilizing the two-sample MR method. Our results demonstrated that ALS was not causally associated with osteoporosis (FN-BMD, LS-BMD, and forearm BMD). In other words, ALS was not an immediate risk factor for osteoporosis. No heterogeneity or pleiotropy existed in the sensitivity analyses, indicating relatively strong robustness of the causal-effect inference in the present study.

Contrary to our findings, several observational epidemiological studies reported robust associations between ALS and osteoporosis, but their findings were contradictory. As far back as 1977, abnormalities in vertebral structure have been observed among ALS patients (Mallette et al., 1977). Subsequently, a significant decrease in cortical bone mass was detected in individuals with ALS (Yanagihara et al., 1984). Likewise, Morini et al. (2023) found that patients with ALS (16 male and 22 female individuals) showed lower BMD. According to a recent cross-sectional study, ALS patients represented statistically significantly worse bone quality parameters (including *T*-score and BMD) compared with the control group matched in sex and age (Caplliure-Llopis et al., 2020). Interestingly, in this series, when analyzing BMD according to different sexes, female patients with ALS (11/12) showed statistically significantly lower BMD than healthy female individuals (4/24); however, differences in the proportion of male individuals with osteoporosis in the ALS group and healthy control group (7/21 vs. 23/42) were not significant. In contrast, one Indian cohort including 30 male ALS subjects and 33 healthy controls suggested a higher level of bone turnover marker in the ALS group; nevertheless, no significant differences in BMD between the two groups were observed (Sooragonda et al., 2021). Yet, all of the above studies were limited by a relatively limited sample size; the associations identified in the observational studies may have been generated by confounders, reverse causality, and a variety of biases, such as selection bias and recall bias. Moreover, inaccurate causal-effect inferences of exposure and outcome still remain, even through rigorous study design and statistical adjustment (Fewell et al., 2007; Davey Smith and Hemani, 2014). Elucidating the causal relationship between ALS and osteoporosis was clinically important.

The mechanism between ALS and osteoporosis has also been widely discussed. Some studies suggest that neurotoxic metals that have been detected in individuals with ALS might have an effect on bone mineralization (Roos et al., 2013). Specifically, evidence from Roos (2014) showed that several neurotoxic metals (e.g., cadmium, lead, and arsenic) accumulate in the bones of ALS patients to disrupt bone remodeling by replacing calcium in hydroxyapatite (one important mineral component of bone). While Ko et al. (2018) revealed that SOD1^G93A^ mice, the mouse model of ALS, exhibited decreased trabecular bone mass, thinner trabeculae, and lower cortical bone thickness when compared with healthy controls. Similarly, by using the G93A mouse model, Zhu et al. (2015) found that mice with serious muscle atrophy showed significantly lower trabecular as well as cortical bone mass. They also found that osteoblast properties, such as osteoblast differentiation capacity, were seriously impaired and that osteoclast formation was markedly improved in the G93A mouse model with serious muscle atrophy. But no striking changes in osteoblast and osteoclast properties were detected in G93A mice without muscle atrophy. Consistently, significant degradation of bone caused by muscle paralysis was found in the murine model (Warner et al., 2006). These animal studies might suggest that muscle atrophy was a direct contributor to osteoporosis, not due to ALS itself.

Accumulative evidence indicated that skeletal muscle load was the major source of mechanical stimulation of bone anabolism; moreover, skeletal muscles were the crucial source of osteogenic growth factors (Hamrick et al., 2010). Clinically, mechanical unloading could lead to bone loss (Lloyd et al., 2014). For instance, one previous study indicated that individuals affected by Duchenne muscular dystrophy showed lower BMD and *Z*-scores (Rufo et al., 2011). Insufficient physical activity was also regarded as a risk factor for osteoporosis (Roos, 2014). Likewise, ALS is a neurodegenerative disease characterized by progressive weakness of skeletal muscles, muscle atrophy, and behavioral deficits. These findings indicated that the presence of osteoporosis in individuals with ALS might be partially intermediated by other elements, including muscle atrophy and physical inactivity. The results of our two-sample MR study revealed no causal relationship between ALS and osteoporosis, which might also suggest an indirect effect, rather than a direct effect, of ALS on osteoporosis.

This study has several strengths. First, our MR study revealed no causal effect of ALS on osteoporosis for the first time at the genetic level, which contributes positively to the genetics of osteoporosis. We further discussed how deteriorated bone health in ALS patients might be due to muscle atrophy or physical inactivity. Based on our results, we recommended that more clinical attention should be paid to the bone health of ALS patients with muscular dystrophy, but osteoporosis should not be dogmatically viewed as a complication of ALS, as our results revealed that ALS may not be causally associated with osteoporosis. The insightful realization that there was no causal association between ALS and osteoporosis helped clinicians place the focus of intervening in osteoporosis in patients with ALS on regular monitoring of bone mineral density and appropriate strategies to nourish the skeletal-muscular system, as well as to enhance muscle contraction and physical activity. Second, the data used in this study were extracted from two large-scale GWAS summary datasets for ALS and osteoporosis separately, which helped to estimate the causality more precisely compared with previous observational studies. Third, there is no significant sample overlap between exposure and outcome datasets in this two-sample MR-designed research. Fourth, BMD in three common bone sites (FN-BMD, LS-BMD, and forearm BMD) was employed as a phenotype for osteoporosis, which might control the statistical bias to some extent. Fifth, no positive findings in the sensitivity analyses excluded the possibility that the MR study was biased by horizontal pleiotropy.

This study has certain limitations. First, considering different bone qualities have been identified between male and female patients with ALS in previous observational studies (Caplliure-Llopis et al., 2020), stratified analysis according to different sexes would have been of clinically great interest. Nevertheless, since we only adopted summary-level databases that are publicly available for MR analyses, it is impossible to perform subgroup analyses. Second, all the participants were of European ancestry; in our study, we did not verify whether a causal effect existed in other populations. Further studies validating the causality among other ethnic groups are needed.

## 5 Conclusion

Contrary to previous observational studies, our study figured out that no causal effect existed between ALS and osteoporosis. The disparity in results is probably attributed to secondary effects such as physical inactivity and muscle atrophy caused by ALS.

## Data availability statement

The original contributions presented in the study are included in the article/Supplementary material, further inquiries can be directed to the corresponding authors.

## Ethics statement

This study is secondary data analysis of publicly available datasets. The original studies involving human participants were reviewed and approved by their relevant Ethics Committees and Review Boards. Written informed consent to participate in the original study was provided by the participants.

## Author contributions

JL: Data curation, Investigation, Methodology, Software, Validation, Writing – original draft. CM: Investigation, Methodology, Software, Writing – review & editing. HH: Investigation, Methodology, Supervision, Writing – review & editing. HL: Conceptualization, Investigation, Methodology, Supervision, Writing – review & editing.

## Footnotes

^1^ https://www.projectmine.com/research/download-data/

^2^ https://sb452.shinyapps.io/overlap/

^3^ https://snipa.helmholtz-muenchen.de/snipa3/

## Glossary

ALS: Amyotrophic lateral sclerosis
BMD: Bone mineral density
CI: Confidence interval
DXA: Dual energy X-ray absorptiometry
FN: Femoral neck
GEFOS: Genetic factors for osteoporosis consortium
GWAS: Genome-wide association studies
IVs: Instrumental variables
IVW: Inverse variance weighted
LD: Linkage disequilibrium
LS: Lumbar spine
MAF: Minor allele frequency
MR: Mendelian randomization
MR-PRESSO: MR pleiotropy residual sum and outlier test
NIMV: Non-invasive mechanical ventilation
OR: Odds ratio
SE: Standard error
SNP: Single nucleotide polymorphism
