# Supplementary figures and images for: Amyotrophic lateral sclerosis and osteoporosis: a two-sample Mendelian randomization study
Source: Front Aging Neurosci. 2023 Dec 14;15:1305040. doi: 10.3389/fnagi.2023.1305040 (PMC10757610; doi:10.3389/fnagi.2023.1305040)

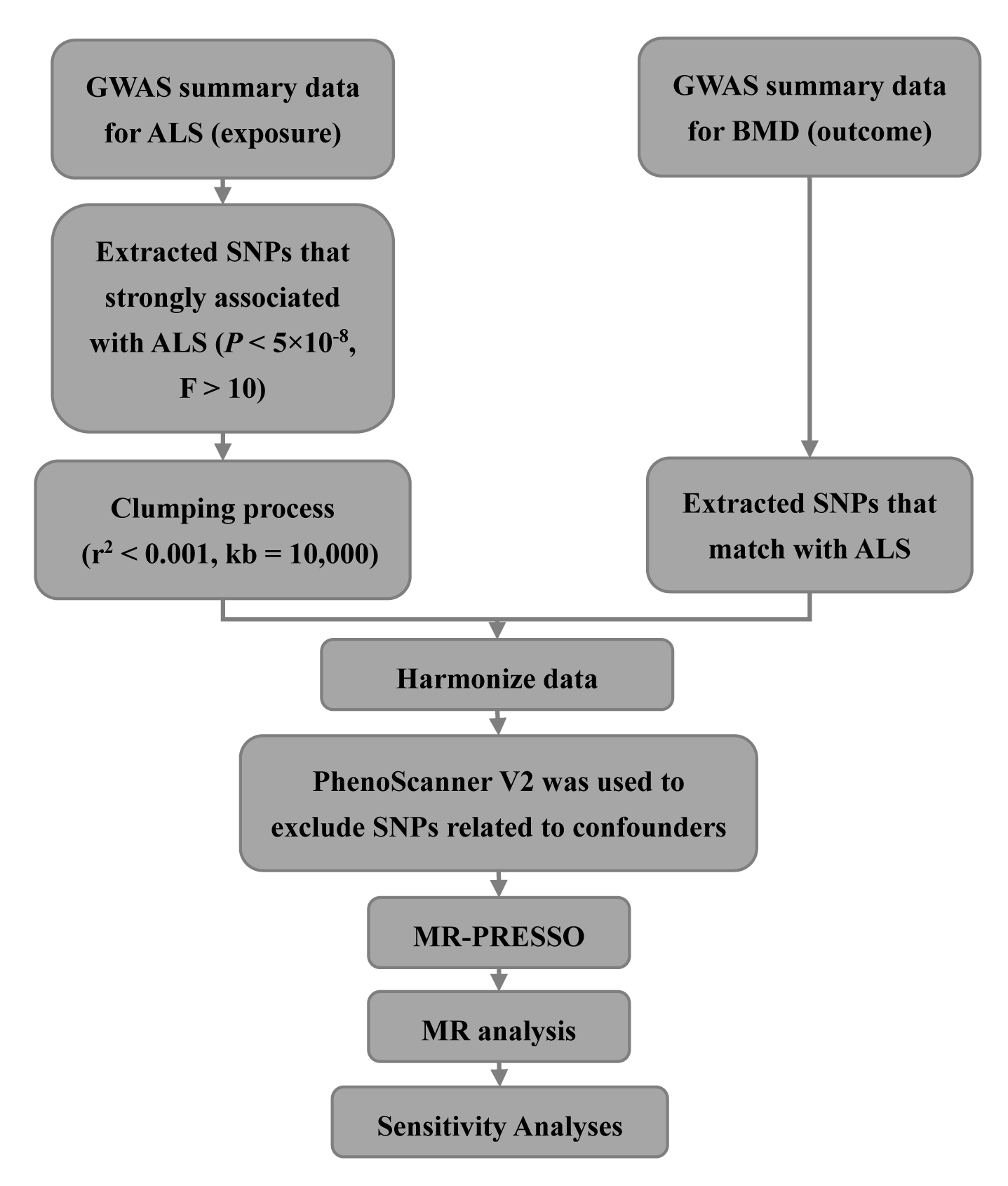

Supplement: Supplementary file 1 [file Image_1.TIF]

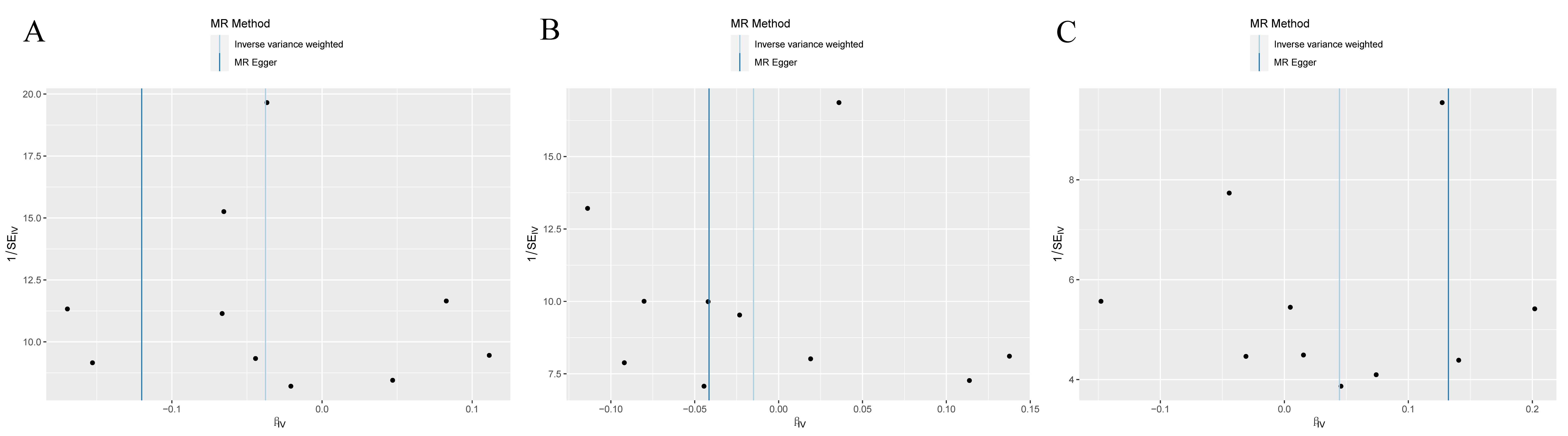

Supplement: Supplementary file 2 [file Image_2.TIF]
