# Supplementary material for: Amyotrophic lateral sclerosis and osteoporosis: a two-sample Mendelian randomization study
Source: Front Aging Neurosci. 2023 Dec 14;15:1305040. doi: 10.3389/fnagi.2023.1305040 (PMC10757610; doi:10.3389/fnagi.2023.1305040)
Supplement: Supplementary file 3 [file Table_1.DOCX]

**Supplemental Table 1** The detailed information of qualified SNPs selected as proxies for ALS.

| SNP | effect_allele | other_allele | beta.exposure | beta.outcome | se.outcome | se.exposure |
| --- | --- | --- | --- | --- | --- | --- |
| rs113247976 | T | C | 0.3322 | -0.056338 | 0.029322 | 0.0492 |
| rs75087725 | A | C | 0.4179 | -0.027798 | 0.037498 | 0.0627 |
| rs10463311 | T | C | -0.0792 | 0.012109 | 0.008649 | 0.0126 |
| rs12608932 | A | C | -0.1247 | 0.008157 | 0.008173 | 0.0121 |
| rs17785991 | A | T | 0.0738 | -0.003268 | 0.00791 | 0.0118 |
| rs229243 | A | C | 0.0922 | 0.00763 | 0.007914 | 0.0118 |
| rs2453555 | A | G | 0.1736 | -0.006369 | 0.008832 | 0.0127 |
| rs4075094 | A | T | -0.0984 | 0.002043 | 0.011983 | 0.0175 |
| rs517339 | T | C | 0.0645 | 0.003033 | 0.007632 | 0.0112 |
| rs631312 | A | G | -0.0791 | -0.008809 | 0.008364 | 0.012 |
